# Patients’ perspectives on dialysis decision-making and end-of-life care 

**DOI:** 10.5414/CN109608

**Published:** 2019-01-21

**Authors:** Fahad Saeed, Muhammad Adil Sardar, Sara N. Davison, Haris Murad, Paul R. Duberstein, Timothy Edward Quill

**Affiliations:** 1 Department of Medicine, Division of Nephrology,; 2 Division of Palliative Care University of Rochester School of Medicine and Dentistry, Rochester,; 3 Department of Psychiatry, and Department of Family Medicine and Center for Center for Communication and Disparities Research, University of Rochester School of Medicine and Dentistry, Rochester, NY,; 4 Department of Medicine, Monmouth Medical Center, Long Branch, NJ, USA,; 5 Division of Nephrology & Immunology, University of Alberta, Edmonton, Alberta, Canada, and; 6 Section of Nephrology, Yale School of Medicine, New Haven, CT, USA

**Keywords:** dialysis, decision making, prognosis, end-of-life care

## Abstract

Background: Few studies have explored dialysis patients’ perspectives on dialysis decision-making and end-of-life-care (EoLC) preferences. We surveyed a racially diverse cohort of maintenance dialysis patients in the Cleveland, OH, USA, metropolitan area. Materials and methods: In this cross-sectional study, we administered a 41-item questionnaire to 450 adult chronic dialysis patients. Items assessed patients’ knowledge of their kidney disease as well as their attitudes toward chronic kidney disease (CKD) treatment issues and EoLC issues. Results: The cohort included 67% Blacks, 27% Caucasians, 2.8% Hispanics, and 2.4% others. The response rate was 94% (423/450). Most patients considered it essential to obtain detailed information about their medical condition (80.6%) and prognosis (78.3%). Nearly 19% of respondents regretted their decision to start dialysis. 41% of patients would prefer treatment(s) aimed at relieving pain rather than prolonging life (30.5%), but a majority would want to be resuscitated (55.3%). Only 8.4% reported having a designated healthcare proxy, and 35.7% reported completing a living will. A significant percentage of patients wished to discuss their quality of life (71%), psychosocial and spiritual concerns (50.4%), and end-of-life issues (38%) with their nephrologist. Conclusion: Most dialysis patients wish to have more frequent discussions about their disease, prognosis, and EoLC planning. Findings from this study can inform the design of future interventions.

## Introduction 

The Institute of Medicine has called for research to improve decision-making and end-of-life care (EoLC) in patients with chronic illnesses such as chronic kidney disease (CKD) [1]. Few studies have explored patients’ perspectives on dialysis decisions and EoLC, even though nearly 25% of dialysis patients die each year and rates of dialysis withdrawal have been increasing [1, 2, 3]. To improve dialysis decision-making and EoLC, we need a better understanding of patients’ preferences on these issues. Specifically, we need data on patients’ knowledge about their disease, their need for prognostic information, satisfaction with dialysis decision making, and advance care planning (ACP). 

Past studies have yielded inconsistent results and did not include representative numbers of African American patients. A study from the University of Alberta showed that nearly 90% of CKD patients wanted detailed information about their medical condition, including prognosis, but fewer than 10% reported having prognostic discussions with their doctors during the prior year [4]. In contrast, a study at the Mayo Clinic of 128 patients, 88% of dialysis patients felt informed or well informed about their prognosis [5]. Whereas the rate of dialysis decisional regret was nearly 61% in one study [4], rates hovered ~ 7% in two others [5, 6]. To the best of our knowledge, the largest recent American study concerning EoLC and dialysis treatment issues in minority patients included only 63 African American patients [7]. To address this gap, large scale US-based studies are needed that include a larger proportion of African American patients. 

The Renal Physicians Association clinical practice guidelines recommend that nephrologists perform ACP with their patients [8], but some primary care physicians may feel responsible to engage in ACP with dialysis patients as part of their overarching patient-care responsibilities [9]. To seek clarification of these responsibilities, it is important to ask patients with CKD about their preferences for these discussions. 

We conducted this study to clarify patients’ perspectives on dialysis treatment decisions and EoLC in a large multi-ethnic sample. Our goal was to explore opportunities to improve the care of these patients. 

## Materials and methods 

We adapted the survey administered for this study from Davison which included patients with CKD stages 4 and 5, and patients receiving dialysis [4]. It was deemed acceptable to adopt the survey questions from Davison’s study since both the studies shared similar objectives despite the difference in demographics and geography [4]. We did not perform a sample size calculation. Before initiating the study, ten physicians and seven staff members (nurses, technologists, and administrators) read the instrument and provided feedback on readability. We then modified the instrument and pilot tested it in a convenience sample of dialysis patients who agreed that no significant changes were necessary. We subsequently distributed it to all adult dialysis patients seen in Ohio Renal Care from November 2014 to December 2014. All dialysis patients under the care of nephrologists affiliated with the Cleveland Clinic were considered eligible to participate and were approached by the second author (MAS). Patients lacking capacity as assessed by the second author were excluded from the study. The second author explained the purpose of this study to the remaining patients and assured them that their responses would remain anonymous and that there would be no effect on their clinical care. Patients who agreed to complete the survey were presumed to have assented to participate. Patients had the option of completing the instrument on-site or at home: patients opting to complete the questionnaire at home had the opportunity to clarify any question during their subsequent dialysis sessions, and patients who had vision problems were able to complete the survey orally on-site. We used descriptive statistics to capture patients’ characteristics and preferences. We categorized all the positive responses (very/somewhat informed) in one category, negative responses (very/somewhat Uninformed) in the second and unsure/no response in the third category. The Cleveland Clinic Institutional Board Review approved this research study. 

## Results 

Out of 450 dialysis patients with capacity to consent, 423 patients participated (94% response rate). 22% (n = 95) completed the survey during a hospitalization. Table 1 shows patients’ characteristics: the majority were African American (67.4%), and 27% were Caucasian. Table 2 shows patients’ self-reported knowledge about their disease, prognosis, and palliative options. 53% reported being “very” or “somewhat” informed about their medical condition, and 27% felt uninformed about their disease. About half were optimistic that their health would improve over the next 12 months, and most reported that they knew about hospice (82%), and 23.4% reported familiarity with palliative care (PC) services. 

Table 3 summarizes patients’ views about prognosis, EoLC planning, and quality of life (QoL) issues. Most patients wanted to know about their prognosis (78%) and most felt it was essential to receive detailed information about their medical condition (80%). 62% expressed a desire to be informed about the full range of treatment options including potentially withdrawing from dialysis, and 74% considered it essential to plan in advance for their death. In making these decisions, two-thirds wanted active family involvement in their medical decision making. Over 70% wanted their nephrologists to discuss their QoL issues with them, and 82% considered QoL to be an essential determinant of their future medical care. Most desired their nephrologists to take care of their physical symptoms (75.7%), and half wanted their nephrologists to help address their spiritual, social, and psychological concerns. 75% rated alternative ways to manage physical symptoms (e.g., holistic care) as very important. 

When asked about the decision whether or not to start dialysis, 45% said they initiated dialysis because of their doctor’s recommendation, and 38% said they made this decision on their own; 19% expressed regret for starting dialysis (Table 4). Over 80% relied on their families for emotional support during their illness and would want their family members to make a medical decision for them if they become incapacitated. Most (91.5%) named their doctors as the primary source of information about their disease. 

Most (67%) respondents had thought about how their illness might progress in the future and many (47%) reported talking about potential life-sustaining treatment options (cardiopulmonary resuscitation or CPR, intubation, etc), but relatively few (5%) had these conversations with their nephrologist and 11% reported talking with their doctors about life expectancy. More patients wanted to have end-of-life discussions with their nephrologists than with their primary care provider (38 versus 25%, p = 0.001). 

36% had completed a living will and 19% had completed a Medical Order for Life-Sustaining Treatments (MOLST) form. 21% had a power of attorney (agent for financial matters), and only 8% had a designated healthcare proxy. If given a choice during their current course of illness, 41% of patients would opt for a treatment plan focused on relieving pain and 31% would prefer life-prolongation care that could entail more pain and suffering. Most patients would prefer to die at home (52.3%) with the support of palliative care. Only 12% of patients wished to die at a hospital, and 17% wanted to die in a hospice facility. 

## Discussion 

In this study of American patients receiving dialysis, most respondents considered it essential to obtain detailed information about their medical condition and prognosis. Dialysis decisional regret was reported by 19% of the respondents. Almost half wanted their nephrologists to be responsive to their spiritual, social and psychological concerns, and more patients wanted to have end-of-life discussions with their nephrologist rather than their primary care physician. 

Most dialysis patients wished to receive detailed medical information, and over a quarter felt uninformed about their kidney disease. Prior research suggests that most patients feel underprepared to start dialysis [10]. In one study, only 50% of CKD patients knew about renal replacement therapy options such as hemodialysis, continuous ambulatory dialysis, and transplant [11]. In another study, only 1% of CKD patients recalled a discussion about conservative CKD management [10]. These findings may be explained in part by the lack of robust CKD education programs and decision-support interventions, but patients’ low educational attainment, variations in practice styles of physicians, time constraints of a patient visit, and limited reimbursement incentives probably also contribute [12, 13]. 

Studies from cancer patients have shown that providing prognostic information helps patients strengthen their relationship with their doctor, and assists in determining their future life and end-of-life goals while simultaneously enhancing hope [14]. According to the United States Renal Data System (USRDS) 2009 report, the average life expectancy of dialysis patients 60 – 64 years of age is ~ 4.5 years [15, 16]. Even though most patients in this study expressed a preference for prognostic discussions, only 11% reported having such discussions with their nephrologists. Nearly half appeared to have a poor understanding of their prognosis; many mistakenly believed that their health would improve over the next 12 months. Adding a question on prognostic delivery on the ESRD Medical Evidence Report Medicare Entitlement and/or Patient Registration Form (Form 2728) could help patients obtain prognostic information. Like cancer patients [17], most dialysis patients are more optimistic than their physicians about their prognosis [18]. Out of fear of diminishing hope, nephrologists might avoid talking about prognosis with patients [14], but this pattern of avoidance could lead many patients to regret beginning dialysis [6]. 

Nearly 4 in 10 patients wanted to have end-of-life discussions with their nephrologist, but few had done so. The need for goals-of-care conversations and ACP is heightened by the fact that patients’ preferences are often not known to their nephrologists or family members [19]. Potential reasons for such low rates of ACP could be nephrologists’ lack of training in such communication [20] and patients and nephrologists’ reluctance to initiate ACP discussions [21]. Differences in individual and cultural beliefs can be associated with lower rates of ACP, but most respondents in this survey reported in theory at least being comfortable having these discussions [7, 22]. 

Patients on dialysis have a high symptom burden including chronic pain, depression, fatigue, and pruritus as well as a shortened life-expectancy [23]. When asked to choose between two mutually exclusive options, most patients wanted an approach that focused on improving QoL (40.6%) as compared to extending life (30.5%), emphasizing the relative importance of symptom management in these patients. Most patients reported that they knew about hospice, but few knew about palliative care. In Davison’s study [4], rates of self-reported patient knowledge about palliative care (12%) was also lower than hospice (22%), and overall knowledge rates were even lower than our study. More systematic integration of palliative care services in dialysis patients could assist with symptom management, potentially improving patients’ QoL [24]. But it will be important to overcome the many barriers to the underutilization of these services [25, 26], including low levels of knowledge of hospice and palliative care services among patients, religious beliefs, fatalism (a concept that everything is pre-decided), physician discomfort with discussing palliative care referral [27], lack of education about palliative care in nephrology fellowships, and financial disincentives [20, 28, 29]. Basic palliative care skills can be acquired by attending continued medical education accredited workshops [30]. However, to observe a substantial change in clinical practice style, a more rigorous primary palliative care curriculum across all the nephrology fellowship programs is necessary [29]. Another consideration could be to offer additional financial incentives to nephrologists to engage in goals of care (GOC) conversations and end-of-life planning in dialysis units. 

Our study has several strengths. The response rate was high and African Americans were well-represented. Our study has several limitations as well. Despite the presence of facilitators to explain the questionnaire to patients, there was still a potential for misunderstanding of our questions. Patient knowledge about hospice and palliative care and presence of advance directives is based on self-report, which is inherently prone to errors. 

In summary, most dialysis patients wish to receive more detailed disease-related knowledge including prognostic information. A majority of these patients want to participate in ACP, and a significant proportion want engagement of nephrologists in addressing their psychosocial and EoLC needs. 

## Funding 

Dr. Saeed is a Mentored Career Development Program (KL2) scholar at the University of Rochester School of Medicine and Dentistry and an awardee of the Geriatric Scholars Award from the University of Rochester, Division of Geriatrics. 

## Conflict of interest 

None. 

**Table 1.** Patient characteristics, n (%).

Characteristics	
Age, years, mean (SD)	59.60 (13.76)
Men	242 (57.2)
Type of treatment	
Hemodialysis	387 (91.5)
Peritoneal dialysis	19 (4.5)
No response	17 (4)
Months on dialysis, mean	
(median, Interquartile range)	50.2 (35,53.5)
Marital status	
Married	163 (38.5)
Single, unmarried, divorced, widowed	259 (61.3)
No response	1 (0.2)
Education	
Less than high school	27 (6.4)
High school	195 (46.1)
Trade school/Technical school	88 (20.8)
University	54 (12.8)
Graduate/Post-graduate	54 (12.8)
No response	5 (1.20)
Religion	
Christianity	363 (85.8)
Judaism	14 (3.3)
Islam	8 (1.9)
Hinduism	3 (0.7)
Atheism	3 (0.7)
Buddhism	1 (0.2)
Don’t believe in God	17 (4.0)
No response	14 (3.3)
Race	
Caucasian	114 (27)
African American	285 (67.4)
Hispanic	12 (2.8)
Native American	5 (1.2)
Other	5 (1.2)

**Table 2.** Patients’ knowledge of their illness and palliative care services, n (%).

How informed are you in regards to your medical condition and how it will change over time?	
Very/Somewhat informed	226 (53.4)
Very/Somewhat Uninformed	112 (26.5)
Unsure/No response	85 (20.1)
How do you see your health in the next 12 months?	
Worsening slightly	37 (8.7)
No change	146 (34.5)
Improving	223 (52.7)
Getting worse	14 (3.3)
No response	3 (0.7)
Do you know what palliative care is?	
Yes	99 (23.4)
No	237 (56)
Unsure/No response	87 (20.5)
Do you know what hospice is?	
Yes	347 (82)
No	35 (8.3)
Unsure/No response	41 (9.6)

**Table 3.** Patient perspective on prognosis, end-of-life care, and quality of life, n (%).

Question	Important	Unimportant	Unsure/No response
How important is it for your "quality of life" to affect your future care?	347 (82)	54 (12.8)	22 (5.2)
How important is detailed information about your medical condition?	341 (80.6)	67 (15.8)	15 (3.6)
How important is it for you to be informed about your prognosis?	331 (78.3)	71 (16.8)	21 (4.9)
How important is it for you to prepare and plan ahead in case of death?	312 (73.7)	69 (16.3)	42 (9.9)
How important is it to you for your family to be actively involved in medical decision making?	318 (75.2)	88 (20.8)	17 (4)
How important is it to you to have access to information on alternative ways to manage your physical symptoms (e.g., holistic care, etc.)	317 (75.2)	61 (14.4)	44 (10.4)
How important is it for you to have your physical symptoms (e.g., pain, nausea) treated by the nephrology staff?	320 (75.7)	66 (15.6)	37 (8.7)
How important is it for you to discuss your “quality of life’’ regularly with our nephrology staff?	301 (71.1)	79 (18.7)	43 (10.2)
How important is it for you to be informed about treatment options such as withdrawing dialysis?	262 (61.9)	105 (24.8)	56 (13.3)
How important is it for you to have your social, psychological, or spiritual concerns attended to by nephrology staff?	213 (50.4)	135 (31.9)	75 (17.7)

**Table 4.** Patients’ end-of-life care preferences, n (%).

Who do you rely on for social and emotional support during your illness and treatment?*	
Family/friends	360 (85.1)
Doctor	54 (12.7)
Nurse	32 (7.6)
Hospital support counselor	19 (4.5)
Spiritual advisor	24 (5.7)
Others	25 (5.9)
If you are physically or mentally unable to make decisions for yourself, who would you choose to make medical decisions for you?*	
Family/friends	363 (85.8)
Doctor	35 (8.3)
Nurse	14 (3.3)
Hospital support counselor	7 (1.6)
Spiritual advisor	9 (2.1)
Others	20 (4.7)
How do you normally get information that will help you make a personal decision about your health/well-being?*	
Specialist (e.g., kidney doctor)?	242 (57.2)
Family physician	145 (34.3)
Family/friends	103 (24.3)
Paper resources	27 (6.4)
Internet	45 (10.6)
Media/TV	14 (3.3)
Other	19 (4.5)
If you are currently receiving dialysis, why did you choose dialysis over conservative care (no dialysis)?	
Your doctor’s wish	190 (44.9)
Your own personal wish	162 (38.3)
Your family’s wish	32 (7.6)
Don’t know/No response	39 (9.2)
If you are currently receiving dialysis, do you regret the decision to start dialysis?	
Yes	80 (18.9)
No	315 (74.5)
Don’t know/No response	28 (6.6)
Are you comfortable discussing end-of-life care issues with your family members?	
Yes	300 (70.9)
No	39 (9.2)
Don’t know/No response	84 (19.8)
Are you comfortable discussing end-of-life care issues with the nephrology staff?	
Yes	267 (63.1)
No	72 (17)
Don’t know/No response	84 (19.9)
Have you thought about what might happen with your illness in the future?	
Yes	284 (67.1)
No	119 (28.1)
Don’t know/No response	20 (4.7)
Has your doctor talked to you about how much time you have to live?	
Yes	45 (10.6)
No	352 (83.2)
Don’t know/No response	26 (6.1)
During the past 12 months, have you had a discussion with any of the following people about your choices concerning end-of-life care?*	
I have not had a discussion about these matters during the last 12 months.	222 (52.5)
Family member	144 (34)
Friend	40 (9.5)
Family doctor	16 (3.8)
Kidney doctor (nephrologist)	23 (5.4)
Nurse or another person from healthcare team	9 (2.1)
Social worker from dialysis unit	15 (3.5)
Spiritual advisor	7 (1.6)
Hospital Support counselor	7 (1.6)
Others	8 (1.9)
Have you completed any of the following?*	
Living will	151 (35.7)
Personal directive/MOLST	82 (19.4)
Healthcare agent/proxy	36 (8.5)
Enduring power of attorney	91 (21.5)
None of the above/Don’t know	195 (46.1)
If you have completed an advance directive, what would you like to be done in case your heart stopped beating?	
Resuscitate (full code)	234 (55.3)
Do not resuscitate	37 (8.7)
Do not know/No response	152 (35.9)
There are a number of things doctors can do to try to revive someone whose heart has stopped beating, which usually includes a machine to help to breathe. Thinking of your current condition, what would you want your doctor to do if your heart stopped beating?	
Restart my heart, if possible, including using a breathing machine	273 (64.5)
Allow me to die-do not try to restart my heart or use a breathing machine	54 (12.8)
Don’t know/No response	74 (22.7)
If you had to choose at this time, would you prefer a course of treatment that focuses on extending life as much as possible, even if it means prolonging pain and discomfort, or would you want a plan of care that focuses on relieving pain and discomfort?	
Relieve pain or discomfort and improve quality of life as much as possible	172 (40.6)
Live as long as possible	129 (30.5)
No response/Don’t know	122 (28.8)
Where would you prefer to die?	
At home with a visiting palliative care support team	222 (52.4)
In a hospice (palliative care) center	72 (17)
Hospital	50 (11.8)
Nursing home	2 (0.5)
Other	39 (9.2)
Don’t know/No response	38 (8.9)
Which members of the healthcare team would you like to talk with about end-of-life issues?*	
Kidney doctor (nephrologist)	160 (37.8)
Family doctor/Primary care physician	105 (24.8)
Nurse	31 (7.3)
Social worker	38 (9)
Hospital support counselor	24 (5.7)
Spiritual advisor	34 (8)
No one	102 (24.1)
Other	23 (5.4)
When would you like to have these end-of-life conversations?	
When I become seriously ill or when the need arises (as defined by your medical team)	153 (36.2)
When I specifically request it	117 (27.7)
Before I am started on dialysis	22 (5.2)
After I start on dialysis but before becoming ill.	89 (21)
Don’t know/No response.	42 (10)
How often would you like to have your end-of-life care plan reviewed?	
Whenever the need arises	170 (40.2)
Whenever I ask for this plan to be reviewed	109 (25.8)
On a regular basis (i.e., annually, semi-annually)	77 (18.2)
Other	38 (9)
Don’t know/No response	29 (6.9)
Where would you like to have these end-of-life discussions?	
In a clinic	170 (40.2)
While on dialysis but in a private room	127 (30)
While on dialysis	66 (15.6)
Don’t know/No response	60 (14.1)

*n’ is greater than 423 because multiple responses were allowed.
